# ACYP1 Is a Pancancer Prognostic Indicator and Affects the Immune Microenvironment in LIHC

**DOI:** 10.3389/fonc.2022.875097

**Published:** 2022-05-02

**Authors:** Lingyi Zhou, Zheng Fu, Shuai Wang, Jing Jia, Yumeng Cheng, Yunxiang Zheng, Ningning Zhang, Wei Lu, Zhi Yao

**Affiliations:** ^1^Department of Immunology, Key Laboratory of Immune Microenvironment and Disease of the Educational Ministry of China, Tianjin Key Laboratory of Cellular and Molecular Immunology, School of Basic Medical Sciences, Tianjin Medical University, Tianjin, China; ^2^Innovation and R&D Management Department, Tianjin Kangzhe Pharmaceutical Technology Development Company, Ltd., Tianjin, China; ^3^Department of Hepatobiliary Oncology, Liver Cancer Center, Tianjin Medical University Cancer Institute and Hospital, National Clinical Research Center for Cancer, Key Laboratory of Cancer Prevention and Therapy, Tianjin Medical University, Tianjin, China; ^4^Department of Clinical Pharmacy, Tianjin Medical University, Tianjin, China; ^5^2011 Collaborative Innovation Center of Tianjin for Medical Epigenetics, Tianjin Medical University, Tianjin, China

**Keywords:** ACYP1, pancancer, liver hepatocellular carcinoma, LIHC, immune infiltration, prognosis, biomarker

## Abstract

**Background:**

ACYP1 plays important physiological and metabolic roles in glycolysis and membrane ion pump activity by catalyzing acyl phosphate hydrolysis. ACYP1 is related to tumorigenesis and progression and poor prognosis in gastrointestinal cancer. However, its pancancer roles and mechanisms are unclear. Our study aimed to understand the ACYP1 expression signature and prognostic value across cancers and investigate immune infiltration patterns in liver hepatocellular carcinoma (LIHC) and verify them in LIHC samples.

**Methods:**

Transcriptional expression profiles of ACYP1 across cancers were analyzed using Oncomine and TIMER. The prognostic value of ACYP1 was assessed across PrognoScan, Kaplan—Meier Plotter, and GEPIA. Significant pathways associated with ACYP1 in LIHC were obtained *via* Gene Set Enrichment Analysis. The correlation between ACYP1 expression and immune infiltration in LIHC was investigated using TIMER. We validated ACYP1 expression, prognostic value, and association with immune cells in tumor tissues by immunohistochemistry and flow cytometry.

**Results:**

ACYP1 was overexpressed across cancers. High expression of ACYP1 correlated with a poor prognosis in most tumor types, especially in LIHC. ACYP1 was significantly implicated in immune and metabolic related pathways. High ACYP1 expression showed significant correlations with the abundances of Th2 cells, Tregs, macrophages, dendritic cells, and myeloid-derived suppressor cells in LIHC. LIHC patients with high ACYP1 expression showed significantly shorter overall survival and relapse-free survival rates concomitant with increased infiltration of CD4+ T cells. Mouse subcutaneous tumors with ACYP1 overexpression exhibited significantly accelerated tumor progression with increased aggregation of CD4+ T cells.

**Conclusion:**

Overall, ACYP1 may serve as a vital prognostic biomarker and play an immunoregulatory role in LIHC.

## Introduction

Acylphosphatase (ACYP) is a small cytoplasmic enzyme commonly found in many organs and tissues of vertebrates. ACYP is involved in multiple cellular physiological processes by catalyzing the hydrolysis of acylphosphates. Compounds containing carboxyl phosphate bonds are substrates of ACYP and play a crucial role in regulating glycolysis, the tricarboxylic acid (TCA) cycle, and membrane ion pump activity (1, 2). Two isoenzymes of ACYP, namely, the erythrocyte and muscle types, have been identified and are encoded by the ACYP1 and ACYP2 genes, respectively. The erythrocyte type has higher catalytic activity. Recent studies have shown that ACYP is involved in erythrocyte senescence and cell differentiation and is also related to metabolism in the context of hyperthyroidism (3–7). In addition, ACYP expression is involved in colon cancer metastasis and glioblastoma progression by regulating intracellular Ca2+ homeostasis (8). Recently, ACYP1 was reported to be involved in imatinib-resistant gastrointestinal stromal tumors (GISTs) (9). ACYP1 is considered to be a metabolism-related gene used to predict the prognosis of patients with gastric and liver cancer and may become an important candidate target for metabolic therapy (10–12). A recent study revealed that the high expression of ACYP1 was significantly associated with a poor prognosis for cholangiocarcinoma (CHOL) patients, which may be related to the effect of ACYP1 on cell viability and apoptosis (13). However, few pancancer studies have been performed on ACYP1, and its mechanism in different tumors remains unclear.

The clinical efficacy of immunotherapy is affected by the tumor microenvironment (TME), which includes hypoxia, low pH, and inhibitory metabolites. Therefore, alleviating metabolic inhibition of immune cells in the TME may improve the efficacy. In general, tumor cells tend to use glycolysis for energy, resulting in the production of large amounts of lactic acid, which inhibits the function of effector T cells by reducing proliferation and cytokine production (14). Several studies have shown that ACYP1 is a metabolism-related gene involved in gastric and liver cancer progression (15–17). ACYP1 can affect glycolysis *via* the dephosphorylation of 1,3 bisphosphoglycerate, and glycolysis was found to be highly active in tissues with high ACYP1 expression (1). However, the role of ACYP1 in the TME of LIHC is still unclear.

This study was focused on the prognostic value across cancers, signaling pathways and the TME in LIHC by analyzing the expression of ACYP1. Immunohistochemical (IHC) and flow cytometry were performed to prove its role in the TME of LIHC. Our study aims to elucidate the importance of ACYP1 in LIHC prognosis.

## Materials and Methods

### ACYP1 Expression and Prognostic Value Across Cancers

In this study, the transcriptional signature of ACYP1 across cancers was analyzed based on the Oncomine database, TIMER database, and UALCAN network. The correlation between levels of ACYP1 mRNA and the ending event of patients with different cancer types was analyzed by assessing PrognoScan (microarray data), Kaplan–Meier Plotter (GEO, TCGA and EGA data) and GEPIA (TCGA and GTEx data) datasets. The effect of ACYP1 mRNA expression on overall survival (OS), disease-free survival (DFS), and relapse-free survival (RFS) across cancers was calculated. The relevance of ACYP1 expression and clinical characteristics of LIHC was determined using the Kaplan–Meier Plotter database.

### GSEA and GSVA Analysis

RNA-seq data were obtained from the TCGA database (https://portal.gdc.cancer.gov/). GSEA and GSVA were performed to explore potential pathways associated with ACYP1 *via* KEGG and Hallmark terms using R version 4.0.5 (normalized enrichment score (NES) ≥1.0 and false discovery rate (FDR) adjusted p value <0.25).

### Correlation Between ACYP1 Expression and Immune Cell Infiltration

The TIMER database used RNA expression profiles to analyze the infiltration of immune cells in tumor tissues. Multiple immune deconvolution methods were used to explore tumor immunological, clinical, and genomic characteristics. The correlation of ACYP1 expression with the abundance of subtypes of immune infiltrating cells, immune cell markers, and tumor purity was analyzed.

### Validation of the Role of ACYP1

Tumor tissue slides of LIHC patients at Tianjin Medical University Cancer Hospital were subjected to IHC using the following antibodies at the indicated concentrations: anti-ACYP1 (ab231323, Abcam) 1:100; anti-CD4 (67786-1-Ig, Peprotech) 1:100; and anti-CD8 (66868-1-Ig, Peprotech) 1:100. Stained tissues were scanned and captured using CaseViewer software (3DHISTECH). The H-scores were used to quantify staining intensity (protein expression). The ACYP1 low expression and high expression groups were set based on the median level of ACYP1 expression. Kaplan—Meier survival curves with log-rank tests were used to analyze the effect of high ACYP1 expression. The use of patient information and tissue was approved by Tianjin Medical University Cancer Institute and the hospital ethics committee.

The ACYP1 protein overexpression plasmids were purchased from Genewiz (Suzhou, China). Briefly, HEK293T cells were transfected with a lentiviral plasmid (Plvx-IRES-Puro-ACYP1) along with packaging plasmids using polyethylenemine. Hep1-6 cells were infected with the virus. Hep1-6 control cells (Vector) and hep1-6 ACYP1 overexpressing cells (ACYP1) were injected into the right flanks of mice (2x10^6^ cells per mouse) to establish a subcutaneous implantation model. From day 8 after injection, tumor size was measured every three days and tumor volume was calculated as length × width^2^/2. After 30 days, the mice were sacrificed to assess tumor growth. The weight of the tumor was also measured. For flow cytometry analysis of immune cell populations, cells from different mouse tumors were divided into an appropriate number of tubes in 100 µl PBS. Cells were stained with antibodies for 30 minutes at 4°C. Absolute cell numbers and frequency of immune cells were identified with appropriate gating. At least 1x10^6^ cells were recorded for tumor analysis. C57BL/6J mice were obtained from Beijing Vital River Laboratory Animal Technologies. All mice were age-matched and kept under specific pathogen-free conditions. All animal procedures were approved by the Animal Ethics Committee of Tianjin Medical University, Tianjin, China.

### Statistical Analysis

All statistics were analyzed using SPSS statistical software (version 24.0.0). P<0.05 was considered to indicate statistical significance. The adjusted P value cutoff of 0.01 and the fold change of 1.5 were set in Oncomine. We used a univariate Cox regression model to calculate the HR and Cox P value and the log-rank test to compare survival curves. Spearman’s correlation was used to analyze the correlation of gene expression.

## Results

### The Expression of ACYP1 in Human Cancers

**Figure 1:**
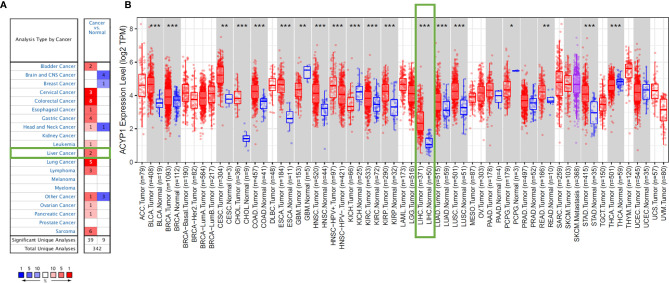
ACYP1 expression levels across cancers. Elevated or decreased levels of ACYP1 expression in multiple cancer types in Oncomine **(A)** and TIMER **(B)**. *P < 0.05, **P < 0.01, ***P < 0.001.

First, we analyzed ACYP1 mRNA expression levels in Oncomine across cancers. ACYP1 expression was higher in a variety of cancer groups than in the respective normal tissues. Decreased expression of ACYP1 was found in the head and neck cancer, breast cancer, etc. (**Figure 1A** and **Supplementary Table S1**). The TIMER database was used to evaluate ACYP1 expression across cancers. ACYP1 expression was significantly higher in most cancers than in their respective adjacent normal tissues (**Figure 1B**). TCGA and GTEx databases were used to identify ACYP1 expression characteristics. ACYP1 was found to be upregulated in lymphoid neoplasm diffuse large B-cell lymphoma (DLBC), pancreatic adenocarcinoma (PAAD), skin cutaneous melanoma (SKCM), thymoma (THYM), etc. ACYP1 was downregulated in some cancers, such as uterine carcinosarcoma (UCS) and brain lower grade glioma (**Supplementary Figure S1**).

### Prognostic Potential of ACYP1 Expression

**Figure 2:**
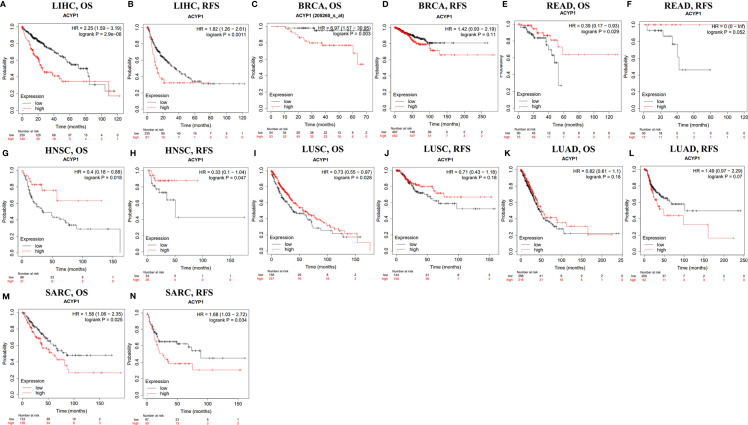
The survival of patients with high and low expression of ACYP1 across cancers in the Kaplan–Meier Plotter database. OS and RFS of liver LIHC **(A, B)**, BRCA **(C, D)**, READ **(E, F)**, HNSC **(G, H)**, LUSC **(I, J)**, LUAD **(K, L)**, and SARC **(M, N)** patients. OS, overall survival; RFS, relapse-free survival.

Based on the difference in expression across cancers, the prognostic value of ACYP1 was predicted in different databases. The relationships between ACYP1 expression levels and prognosis for each type of cancer were explored using Kaplan–Meier Plotter. ACYP1 was found to be an adverse prognostic factor for LIHC (OS: HR=2.25, p=2.9e-0.6; RFS: HR=1.82, p=0.0011) (**Figures 2A, B**). ACYP1 also had an adverse effect on OS, but not on RFS for BRCA patients (OS: HR=6.97, p=0.003) (**Figures 2C, D**). For colorectal cancer, ACYP1 was only a favorable prognostic factor in terms of OS, but not RFS in READ (HR=0.39, p=0.029) (**Figures 2E, F**). For HNSC, ACYP1 was a significant protective factor in terms of OS and RFS (OS: HR=0.4, p=0.018; RFS: HR=0.33, p=0.047) (**Figures 2G, H**). In addition, high expression of ACYP1 was beneficial only for LUSC patients (OS: HR=0.73, p=0.028; RFS: HR=0.71, log-rank p=0.18) (**Figures 2I, J**) and not LUAD patients, in which ACYP1 had an adverse effect on RFS (OS: HR=0.82, p=0.18; RFS: HR=1.49, p=0.07) (**Figures 2K, L**). For soft tissue cancer, ACYP1 significantly influenced OS and RFS (OS: HR=1.58, p=0.025; RFS: HR=1.68, p=0.034) (**Figures 2M, N**).

According to the ACYP1 expression level, patients were divided into two groups to assess the role in survival *via* PrognoScan. The results showed that high ACYP1 expression was an adverse prognostic factor for five of these tumor types, including breast, colorectal, brain, soft tissue cancers and skin. Interestingly, in lung cancer and head and neck cancers, ACYP1 played a protective role (**Supplementary Figures S2A–J**). These results suggested that ACYP1 may play variable roles across cancers, while the role of poor prognosis may be predominant.

We also analyzed the RNA sequencing data of ACYP1 in TCGA by using GEPIA. We first analyzed the overall effect of ACYP1 on all 33 cancer types. Of note, high expression of ACYP1 indicated poor prognosis across cancers in a total of 9497 patients, and OS and DFS were significantly different (OS: HR= 1.1, P = 0.039; DFS: HR= 1.1, P = 0.049) (**Supplementary Figures S3A, B**). We then analyzed the role of ACYP1 in each of the cancer types. However, unlike findings from PrognoScan and Kaplan–Meier Plotter, high expression levels of ACYP1 only indicated a poorer prognosis in LIHC (OS: p=0.00015; RFS: p=0.00026) (**Supplementary Figures S3C–P**).

### Relationship Between ACYP1 Expression and Clinical Features in LIHC Patients

**Figure 3:**
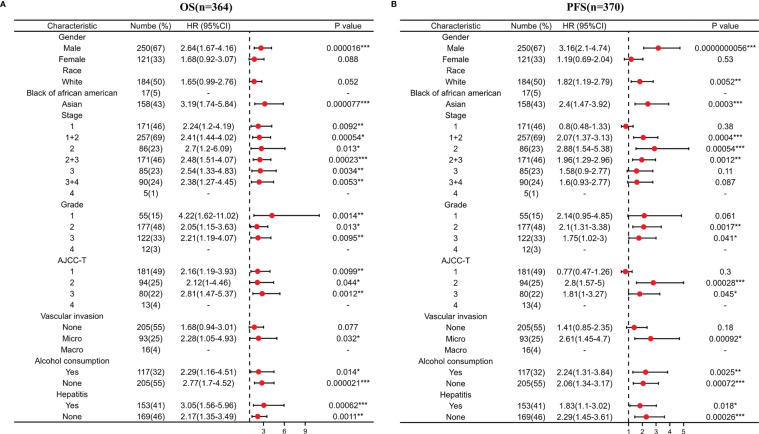
Correlation of ACYP1 expression from LIHC patients in various clinicopathologies with OS (n=364) **(A)** and RFS (n=370) **(B)**. Red dots represent the hazard ratio. OS, overall survival; PFS, progression-free survival. *P < 0.05, **P < 0.01, ***P < 0.001.

Then we investigated the relationship between ACYP1 expression and clinicopathologic characteristics. The results showed that male sex, Asian race, stage I tumor, stage, grade, AJCC T stage, microvascular invasion, history of alcohol consumption, and hepatitis virus infection were closely related to high expression of ACYP1, which indicates a worse OS in LIHC (**Figure 3A**). Similarly, high expression of ACYP1 also indicates a worse PFS in LIHC, especially in males, white race, Asian race, stage I to stage III, grade 2 and 3, AJCC T2 and T3, microvascular invasion, alcohol consumption, and hepatitis virus infection (**Figure 3B**).

The results from the Wurmbach mixed liver dataset showed that ACYP1 expression varied significantly by cancer type, grade, hepatitis virus infection status, and vascular invasion (**Supplementary Figures S4A–D**). In the UALCAN database, ACYP1 expression in cancer stage, patient age, tumor grade, and histological subtypes was significantly different. No association was found between ACYP1 expression and the patient’s race or sex(**Supplementary Figures S4E–J**). Moreover, ACYP1 was significantly associated with early and late recurrence of LIHC. ACYP1 levels were significantly upregulated in patients with early recurrence (**Supplementary Figure S5**). ACYP1 may be an important factor in the recurrence and prognosis of LIHC.

### Significant Pathways Associated With ACYP1 in LIHC

**Figure 4:**
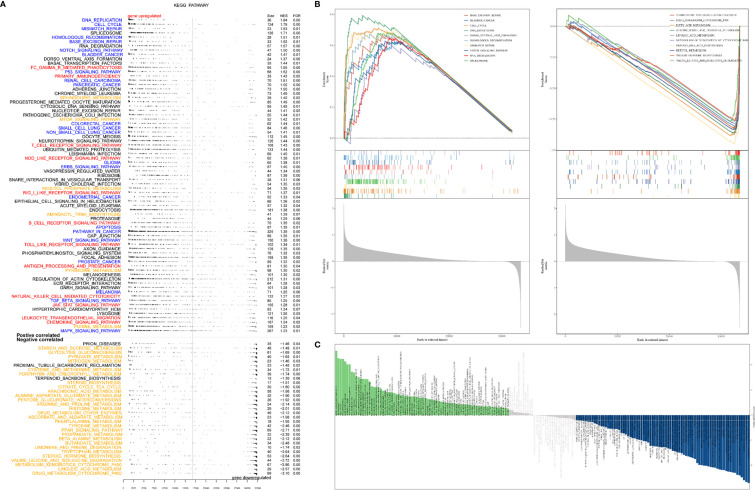
Signal pathways involved in ACYP1. **(A)** Correlation between ACYP1 expression and KEGG pathways in LIHC analyzed *via* GSEA. **(B)** The top ten signaling pathways with significant positive and negative correlations with ACYP1 in LIHC. **(C)** Relationships between ACYP1 expression and KEGG pathways in LIHC performed *via* GSVA (NSE≥1.0, FDR<0.25).

We performed KEGG GSEA and GSVA (**Supplementary Table S2**) to explore the potential role of ACYP1 in signaling pathways. The immune-related pathways were highly enriched in LIHC by KEGG GSEA (NSE≥1.0, FDR<0.25), including Fc-gamma-r-mediated phagocytosis, primary immunodeficiency, TCR signaling pathway, antigen processing and presentation, JAK-STAT pathway, leukocyte transendothelial migration, chemokine signaling pathway and so on, which are shown in red (**Figure 4A**). It also affected several cancer-related pathways, such as DNA replication, the P53 signaling pathway, apoptosis, the WNT signaling pathway, and the MAPK signaling pathway, as shown in blue. ACYP1, as an important enzyme involved in glycolysis (**Supplementary Figure S6D**), was significantly involved in metabolism-related pathways, including glycolysis gluconeogenesis, sphingolipid metabolism, pyruvate metabolism, mTOR signaling, inositol phosphate metabolism, aminoacyl transfer biosynthesis, pyrimidine metabolism, and purine metabolism, as shown in yellow. The top ten signaling pathways related to ACYP1 are shown in **Figure 4B**. The KEGG GSVA analysis showed similar pathways associated with ACYP1 in LIHC (**Figure 4C**). Furthermore, the Hallmark GSEA and GSVA analysis (**Supplementary Table S3**) showed that immune-related pathways, including the TNFα signaling pathway, allograft rejection, inflammatory response, and interferon-gamma response, were highly enriched in LIHC (**Supplementary Figures S6A–C**).

### ACYP1 Expression and Immune Infiltration in LIHC

**Figure 5:**
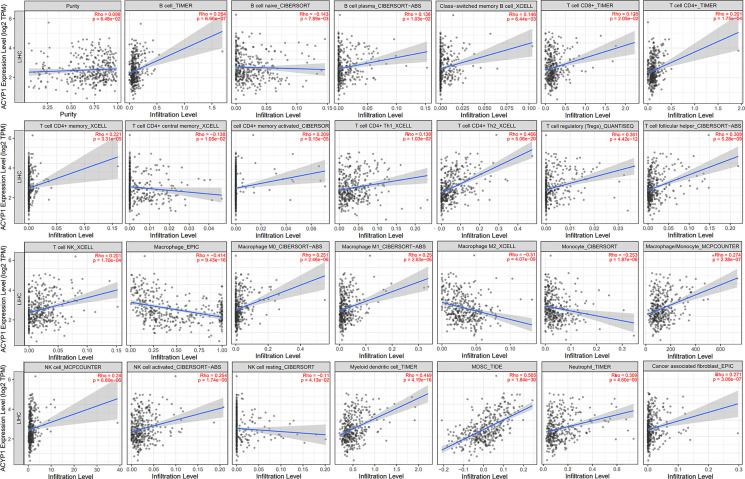
Effects of ACYP1 expression on the TME in LIHC by using TIMER. ACYP1 expression had a significant correlation with the infiltration of various immune cells, including B cells, CD8+ T cells, CD4+ T cells, Tregs, Tfh cells, macrophages, monocytes, macrophages/monocytes, NK cells, DCs, MDSCs, neutrophils, cancer-associated fibroblasts, and their respective subtypes. LIHC, liver hepatocellular carcinoma.

Based on the above analysis, we determined that ACYP1 plays an important role in regulating immune-related pathways in LIHC. Therefore, it is necessary to investigate whether ACYP1 expression is related to immune infiltration in LIHC. The results indicated that ACYP1 expression had no significant correlations with tumor purity. In addition, the ACYP1 expression level was closely related to the infiltration of B cells (R=0.264, p=6.66e-07) and other B-cell subtypes; including CD8+ T cells (R=0.125, p=2.05e-02), CD4+ T cells (R=0.201, p=1.75e-04) and their subtypes, Tregs (R=0.361, p=4.42e-12), NK cells (R=0.201, p=1.70e-04), macrophages (R=-0.414, p=9.43e-16), monocytes (R=-0.253, p=1.87e-06), macrophages/monocytes (R=0.274, p=2.38e-07), dendritic cells (DCs) (R=0.419, p=4.19e-16), myeloid-derived suppressor cells (MDSCs) (R=0.565, p=1.84e-30), neutrophils (R=0.309, p=4.60e-09) and cancer-associated fibroblasts (R=0.271, p=3.06e-07) (**Figure 5**). We created a heatmap and lollipop plot of the correlation between ACYP1 and infiltration of various immune cell types *via* CIBERSORT (**Supplementary Figure S7**, **Supplementary Table S4**). Moreover, correlation analysis of ACYP1 in all other tumors with various immune cell infiltrations was performed in TIMER1.0 (**Supplementary Figure S8**) and TIMER2.0 (**Supplementary Figure S9**), respectively. These results revealed that ACYP1 expression affects patient survival by affecting the infiltration of various immune cell types in LIHC, with the strongest correlations with CD4+ Th2 T cells, Tregs, DCs, macrophages, and MDSCs.

Then the correlation between ACYP1 expression and the expression of biomarkers of immune cell subsets was investigated in LIHC patients. Some representative markers were selected to characterize various infiltrating immune cells in LIHC. We analyzed T-cell subsets, such as CD8+ T cells, Th1 cells, Th17 cells, Tregs, exhausted T cells, and different types of macrophages, such as M1, M2, and tumor-associated macrophages. The results showed that ACYP1 expression was significantly associated with the expression of 45 of the 49 assessed markers in LIHC. Similarly, ACYP1 expression was significantly correlated with the infiltration levels of MDSCs, Th2 cells, DCs, and macrophages and respective markers for these four immune cell types (**Table 1**). Moreover, ACYP1 expression was correlated with the infiltration of B cells, T cells and other subtypes, neutrophils, and NK cells infiltration to varying degrees. Therefore, these results confirmed that ACYP1 expression may influence the infiltration of various immune cell types in different ways and ultimately comprehensively affect the prognosis of LIHC patients.

**Table 1:** Correlation between ACYP1 expression and immune cell markers in LIHC.

Cell type	Gene markers	Cor	*p*	
B cell	CD19	0.238	0.000008	***
	CD79A	0.167	0.001880	**
T cell	CD3D	0.303	0.000000	***
	CD3E	0.24	0.000007	***
	CD2	0.223	0.000029	***
CD8+ T cell	CD8A	0.205	0.000128	***
	CD8B	0.208	0.000099	***
Th1	T-bet	0.096	0.074700	
	IL-2	0.116	0.031700	*
	IFN-γ	0.243	0.000005	***
	TNF	0.272	0.000000	***
Th2	GATA3	0.261	0.000001	***
	STAT5A	0.242	0.000005	***
	IL13	0.178	0.000896	***
Th17	STAT3	0.147	0.006170	**
	IL17A	0.08	0.137000	
Tfh	BCL6	0.212	0.000075	***
	IL21	0.071	0.189000	
Treg	FOXP3	0.214	0.000061	***
	CCR8	0.312	0.000000	***
	STAT5B	0.187	0.000495	***
	TGFβ	0.325	0.000000	***
T cell exhaustion	PD-1	0.237	0.000009	***
	CTLA-4	0.359	0.000000	***
	LAG3	0.274	0.000000	***
	TIM-3	0.379	0.000000	***
	GZMB	0.152	0.004630	**
	TOX	0.228	0.000019	***
	TIGIT	0.305	0.000000	***
TAM	CCL2	0.232	0.000013	***
	CD68	0.205	0.000122	***
	IL10	0.239	0.000007	***
M1 macrophage	iNOS	0.087	0.109000	n.s.
	IRF5	0.366	0.000000	***
	COX2	0.276	0.000000	***
M2 macrophage	CD163	0.109	0.042400	*
	VSIG4	0.185	0.000547	***
	MS4A4A	0.172	0.001380	**
Neutrophils	CD66b	0.094	0.081200	n.s.
	CD11b	0.337	0.000000	***
NK	CD16	0.330	0.000000	***
	CD56	0.346	0.000000	***
DC	CD1c	0.103	0.057100	n.s.
	CD83	0.37	0.000000	***
	CD209	0.104	0.053600	n.s.
	MHCII	0.219	0.000042	***
Monocyte	CD14	-0.301	0.000000	***
	CD64	0.338	0.000000	***
	CD15	0.368	0.000000	***

LIHC, liver hepatocellular carcinoma; Tfh, follicular helper T cell; Th, T helper cell; Treg, regulatory T cell; TAM, tumor associated macrophage; NK, natural killer cell; DC, dendritic cell; Cor, R value of Spearman’s correlation. ^*^P < 0.05, ^**^P < 0.01, ^***^P < 0.001. n.s. "no significance".

### Preliminary Experimental Verification of the ACYP1 Signature in LIHC

**Figure 6:**
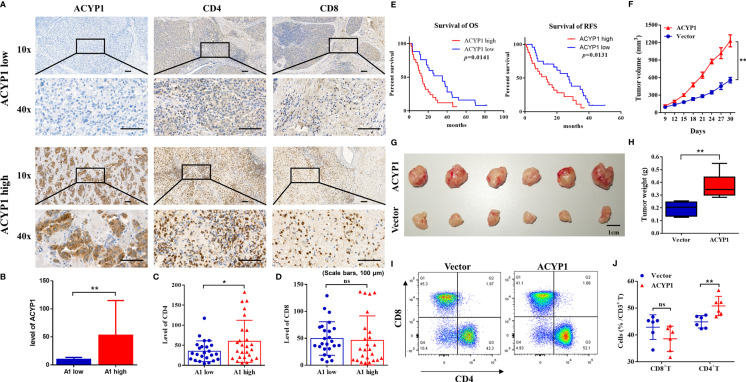
Preliminary experimental verification of the ACYP1 signature in LIHC. **(A)** Representative samples with low and high ACYP1 expression for IHC staining of ACYP1, CD4+ and CD8+ T cells in the ACYP1 low and high group were taken at 100x and 400x magnification. **(B–D)** Relative quantitative analysis of ACYP1 **(B)**, CD4 **(C)** and CD8 **(D)** levels by IHC staining. **(E)** Survival analysis of the ACYP1 high group and the ACYP1 low group in 50 LIHC patients. **(F)** Subcutaneous growth of Hep1-6 tumors in the vector group and ACYP1 overexpression group (n=6). **(G)** Representative images of subcutaneous Hep1-6 tumors in the vector group and ACYP1 overexpression group (n=6). **(H)** The weight of subcutaneous Hep1-6 tumors in vector group and ACYP1 overexpression group (n=6). **(I, J)** The relative content of CD4+ T cells and CD8+ T cells in subcutaneous Hep1-6 tumors from vector mice and ACYP1 overexpressing mice (n=6). Positive cell percentages are shown. *P < 0.05, **P < 0.01, n.s. "no significance".

IHC was performed to evaluate the expression of ACYP1 in 50 LIHC specimens. According to ACYP1 expression level, all specimens were classified as either high expression (ACYP1 high) or low expression (ACYP1 low). Images of representative samples were taken at 100× and 400× magnification and ACYP1 expression is shown (**Figures 6A, B**). Of note, we investigated the prognostic role of ACYP1 in LIHC patients and revealed that patients with high expression of ACYP1 had worse OS (p=0.0141) and RFS (p=0.0131) (**Figure 6E**). The relative levels of immune cell infiltration showed that the infiltration of CD4+ T cells, but not CD8+ T cells was stronger in the ACYP1 high expression group than in the other group (p<0.05) (**Figures 6A–D**). Then, we extended our studies to the subcutaneous implantation tumor model of Hep1-6 (mouse hepatoma cells) to explore the potential roles of ACYP1 in tumor progression and the TME. The expression of ACYP1 was significantly up-regulated in ACYP1-overexpressing stable cell lines (**Supplementary Figure S10A**). Remarkably, ACYP1 overexpression significantly accelerated tumor growth under the skin (**Figure 6F**). The tumor in the ACYP1 overexpression group were significantly larger and weighted more than those in the vector group (**Figures 6G, H**). Moreover, we explored whether immune cells were involved in the high ACYP1 expression-mediated LIHC progression. In comparison with the vector group, a prominent accumulation of CD4+ T cells was found in ACYP1 overexpressing mice, while no obvious changes were found in CD8+ T cells (**Figures 6I, J** and **Supplementary Figure S10B**). These results indicated that ACYP1 expression was positively related to CD4+ T cells, which is consistent with the results from TCGA.

## Discussion

Acylphosphatase, as a cytoplasmic enzyme, widely exists in a variety of vertebrate tissues and can catalyze the hydrolysis of carboxyl phosphate bonds in carboxyl phosphate. These carboxyl phosphate bonds mainly exist in two forms: metabolic products, such as 1,3-bisphosphoglyceric acid, and acetyl phosphate, and membrane ion pump intermediates, such as active intermediates of K+, Ca2+, and Na+-ATPase (18–20). The hydrolytic activity of ACYP on 1,3 diphosphoglyceric acid suggests that ACYP plays a regulatory role in glycolysis. It has been confirmed that ethanol production is significantly increased in yeast cells overexpressing the ACYP gene. ACYP plays a regulatory role in the ion transport system. It affects membrane pumps *via* hydrolyzing the phosphate of aspartic acid under the action of Ca2+, Na+, and K+-ATPase in neuronal erythrocyte membranes, cardiac sarcolemmas, and skeletal muscle sarcolemmas (19–23).

The roles of the ACYP in cancer have not been well studied, and a few studies have explored this topic. ACYP expression is associated with the metastatic phenotype of human colorectal cancer and plays an oncogenic role in gliomas *via* activating the c-MYC signaling pathway (8, 24). Recent studies have shown that high expression of ACYP1 can affect the prognosis of CHOL, and hepatocellular CHOL patients *via* affecting cell proliferation *in vitro* (13).

As a metabolism-related gene with prognostic value in gastric cancer and liver cancer, ACYP1 may become an important candidate target for metabolic therapy (10–12). Our study revealed that ACYP1 plays an adverse role in LIHC patients, although its implication may vary depending on clinical characteristics such as sex, race, tumor stage, tumor grade, vascular invasion, alcohol consumption, and hepatitis status according to multifactor regression analysis. The TME is likely to help elucidate the underlying mechanisms of tumor progression. In this study, ACYP1 overexpression in LIHC tumors may result in much more immune suppressive cell infiltration, while the molecular mechanism needs to be further investigated.

There are some studies have explored biomarkers of prognosis or treatment in pancancer based on public databases. The results suggested that single gene may deeply involve in the immunological features and cancer prognosis. Chao Deng et al. performed multidimensional bioinformatics analysis to examine the relationship between NRP genes and prognostic in pancancer, and finally found the NRP family genes are significantly correlated with cancer prognosis and immune infiltration in bladder urothelial carcinoma (25). Xiaoyu Zhang and colleagues reported MXD3 played an important role in predict prognosis of glioma, and may expected to be as a clinical therapeutic target *via* analyzing public databases and experimental validation (26). To facilitate researchers exploring the prognosis associated genetic features in multi-omic levels in pancancer and finding critical genes for drug discovery and precision medicine, an online platform was developed by Ouyang Jian and colleagues. Our results were further validated on this platform (27). In this study, we analyzed the expression levels of ACYP1 and its prognostic value across cancers using multiple databases. First, in Oncomine, ACYP1 expression was higher in a variety of cancer groups versus their corresponding normal control groups. Moreover, reduced expression of ACYP1 was found in brain cancer, breast cancer, head and neck cancer, and other cancers, while TCGA database analysis results *via* TIMER were not the same. The differences may be due to different algorithms in the two databases. We further explored the effect of ACYP1 on the prognosis of various cancers. In PrognoScan, high ACYP1 expression was a risk factor for adverse outcomes in breast, colorectal, brain, skin, and soft tissue cancers. In Kaplan–Meier Plotter and GEPIA, ACYP1 was closely related to a poor prognosis in LIHC and was related to the following clinical characteristics: male sex, Asian race, all tumor stages, all tumor grades, AJCC T stage, microvascular invasion, alcohol consumption (yes or no), and hepatitis virus infection (yes or no). Thus, at present, we have evidence that ACYP1 has the potential to be a pancancer prognostic biomarker, especially in LIHC.

The TME can inhibit immune responses *via* multiple mechanisms. Aerobic glycolysis, known as the Warburg effect, the most common type of tumor metabolic reprogramming, the results in a TME characterized by hypoxia, low pH, low nutrient status and the production of inhibitory metabolites such as lactic acid, which promote tumor progression by affecting immune cell function. Aerobic glycolysis is a hallmark of liver cancer, which involves cancer progression, and even induces an immunosuppressive TME *via* key enzymes (28). In breast cancer, aerobic glycolysis affects MDSCs and maintains tumor immunosuppression (29). Macrophages promote tumor growth *via* regulating tumor cell aerobic glycolysis with PGK1 phosphorylation (30). The glycolysis pathway is actively regulated during the differentiation of Tregs, providing necessary biological energy sources for Tregs biosynthesis, proliferation, and migration (31–33). Therefore, addressing the metabolic limitations of immune responses in the tumor immune microenvironment may improve the effectiveness of cancer immunotherapy. Given that ACYP1 is an important metabolic enzyme in glycolysis, we hypothesized that the upregulation of ACYP1 in LIHC may affect immune cell infiltration *via* increasing the glycolysis rate. In our study, the pathway analysis suggested that ACYP1 was highly related to immune-related pathways in LIHC. The higher expression of ACYP1 may be closely related to negative immunity in the tumor itself. ACYP1 expression was also significantly correlated with the gene markers of these four immune cell types. The strength of the correlation between different markers and ACYP1 expression varied, suggesting that ACYP1 has characteristic interactions with certain immune cell subtypes.

The results from public data analysis were verified *via* using human LIHC tumor specimens and mouse subcutaneous tumor specimens. First, in LIHC patients, high expression of ACYP1 indicates a poor OS. In a mouse subcutaneous tumor model, high expression of ACYP1 was closely related to tumor progression. Moreover, immune infiltration was assessed by IHC in human tumor specimens and flow cytometry in mouse tumor samples. The results were consistent with the TCGA data, in which high expression of ACYP1 was positively associated with CD4+ T cells, but was not correlated with CD8+ T cells. Our results suggest the possibility that the immunosuppressive TME resulting from by ACYP1 may be the culprits of LIHC progression.

**Figure 7:**
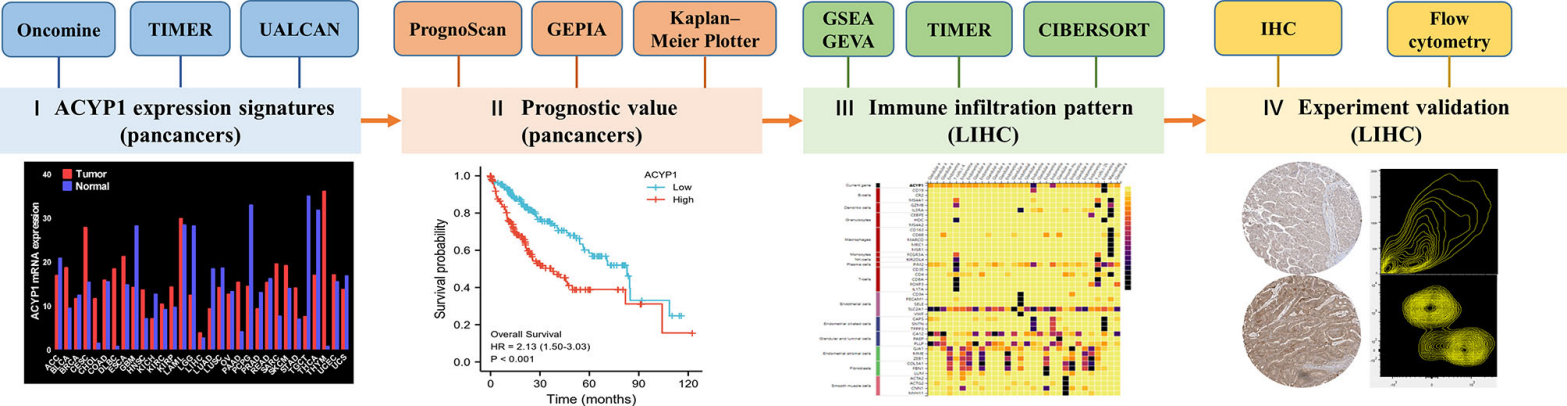
Study flow-chart. I ACYP1 expression patterns explored *via* Oncomine, TIMER, and UALCAN; II Survival analysis of ACYP1 in PrognoScan, Kaplan—Meier Plotter, and GEPIA; III Signaling pathways and TME affected by ACYP1 *via* GSEA, GSVA, and TIMER; IV Experimental validation in human and mice tumor samples of LIHC.

In this study, we investigated the roles of ACYP1 in pancancer and eventually showed an important role in the development of LIHC (**Figure 7**). Based on its physiological role and ectopic expression in liver cancer, our analysis integrated metabolism-related pathways and the immune microenvironment. The results showed that ACYP1 was an enzyme in the progress of glycolysis and promoted tumor progression *via* participating in the glycolysis and immunosuppression. Interestingly, glycolysis was closely associated with immune microenvironment and affect the prognosis of patients. The liver itself is an immune organ, with rich populations of immune cells, and it is also a key organ in regulating glucose metabolism (34). In the process of LIHC development and treatment, changing in metabolism and immune cells play a crucial role. So, a bridge between glucose metabolism and immunotherapy was necessary. According to our results, ACYP1 could be that link, and as a candidate drug target for LIHC intervention.

In conclusion, we demonstrated for the first time the role of ACYP1 in predicting prognosis, and the TME, and associated pathways across cancers. To this end, we confirmed that ACYP1 predicts a poor prognosis and an immunosuppressive TME in LIHC. Overall, ACYP1 has potential as a pancancer prognostic marker from the perspective of tumor immunology and provides a novel target for the treatment of LIHC.

## Data Availability Statement

The datasets presented in this study can be found in online repositories. The names of the repository/repositories and accession number(s) can be found in the article/**Supplementary Material**.

## Ethics Statement

The studies involving human participants were reviewed and approved by Tianjin Medical University Cancer Institute and the hospital ethics Committee. The patients/participants provided their written informed consent to participate in this study. The animal study was reviewed and approved by Animal Ethics Committee of Tianjin Medical University.

## Author Contributions

LZ: Formal analysis, Investigation, Writing - Original Draft. ZF: Methodology, Data Curation. SW: Conceptualization, Software. JJ: Validation, Visualization. YC: Data Curation, Resources. YZ: Resources. NZ: Software, Resources. WL: Supervision. ZY: Writing - Review & Editing, Supervision, Funding acquisition. All authors contributed to the article and approved the submitted version.

## Funding

This work was supported by a grant from the General Program of National Natural Science Foundation of China [82173199].

## Conflict of Interest

Authors ZF and JJ were employed by Tianjin Kangzhe Pharmaceutical Technology Development Company, Ltd.

The remaining authors declare that the research was conducted in the absence of any commercial or financial relationships that could be construed as a potential conflict of interest.

## Publisher’s Note

All claims expressed in this article are solely those of the authors and do not necessarily represent those of their affiliated organizations, or those of the publisher, the editors and the reviewers. Any product that may be evaluated in this article, or claim that may be made by its manufacturer, is not guaranteed or endorsed by the publisher.

## Abbreviations

ACC, Adrenocortical carcinoma; BLCA, Bladder Urothelial Carcinoma; BRCA, Breast invasive carcinoma; CESC, Cervical squamous cell carcinoma and endocervical adenocarcinoma; CHOL, Cholangio carcinoma; COAD, Colon adenocarcinoma; DLBC, Lymphoid Neoplasm Diffuse Large B-cell Lymphoma; ESCA, Esophageal carcinoma; GBM, Glioblastoma multiforme; HNSC, Head and Neck squamous cell carcinoma; KICH, Kidney Chromophobe; KIRC, Kidney renal clear cell carcinoma; KIRP, Kidney renal papillary cell carcinoma; LAML, Acute Myeloid Leukemia; LGG, Brain Lower Grade Glioma; LIHC, Liver hepatocellular carcinoma; LUAD, Lung adenocarcinoma; LUSC, Lung squamous cell carcinoma; MESO, Mesothelioma; OV, Ovarian serous cystadenocarcinoma; PAAD, Pancreatic adenocarcinoma; PCPG, Pheochromocytoma and Paraganglioma; PRAD, Prostate adenocarcinoma; READ, Rectum adenocarcinoma; SARC, Sarcoma; SKCM, Skin Cutaneous Melanoma; STAD, Stomach adenocarcinoma; TGCT, Testicular Germ Cell Tumors; THCA, Thyroid carcinoma; THYM, Thymoma; UCEC, Uterine Corpus Endometrial Carcinoma; UCS, Uterine Carcinosarcoma; UVM, Uveal Melanoma; TME, Tumor Microenvironment; IHC, Immunohistochemical; OS, Overall Survival; DFS, Disease-free Survival; RFS, Relapse-free Survival; HR, Hazard Ratio; GSEA, Gene Set Enrichment Analysis; GSVA, Gene Set Variation Analysis; DCs, Dendritic Cells; MDSCs, Myeloid-derived Suppression Cells.
