# Supplementary material for: ACYP1 Is a Pancancer Prognostic Indicator and Affects the Immune Microenvironment in LIHC
Source: Front Oncol. 2022 May 2;12:875097. doi: 10.3389/fonc.2022.875097 (PMC9108903; doi:10.3389/fonc.2022.875097)
Supplement: Supplementary file 1 [file DataSheet_1.docx]

Supplementary Material

#
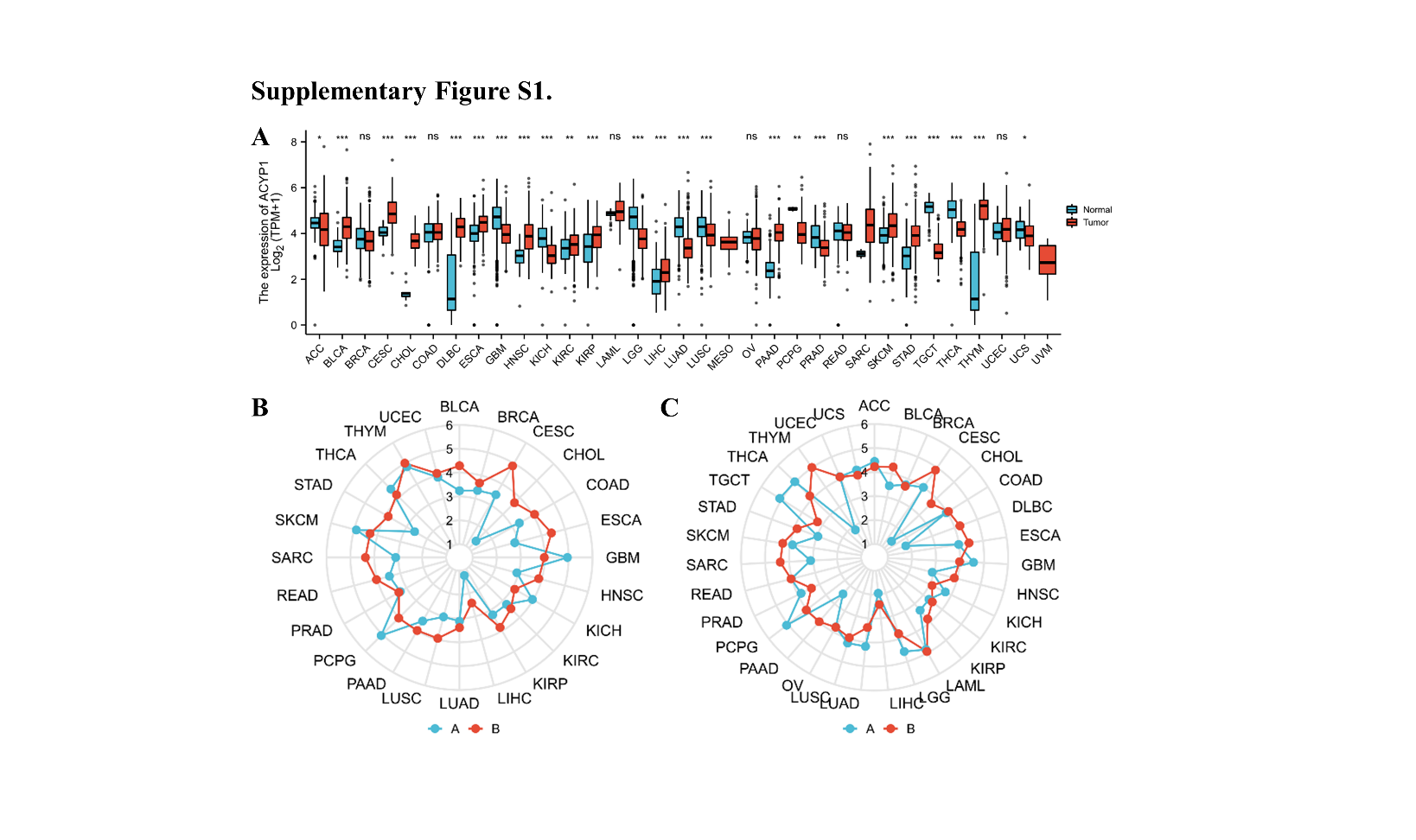
Supplementary Figures:


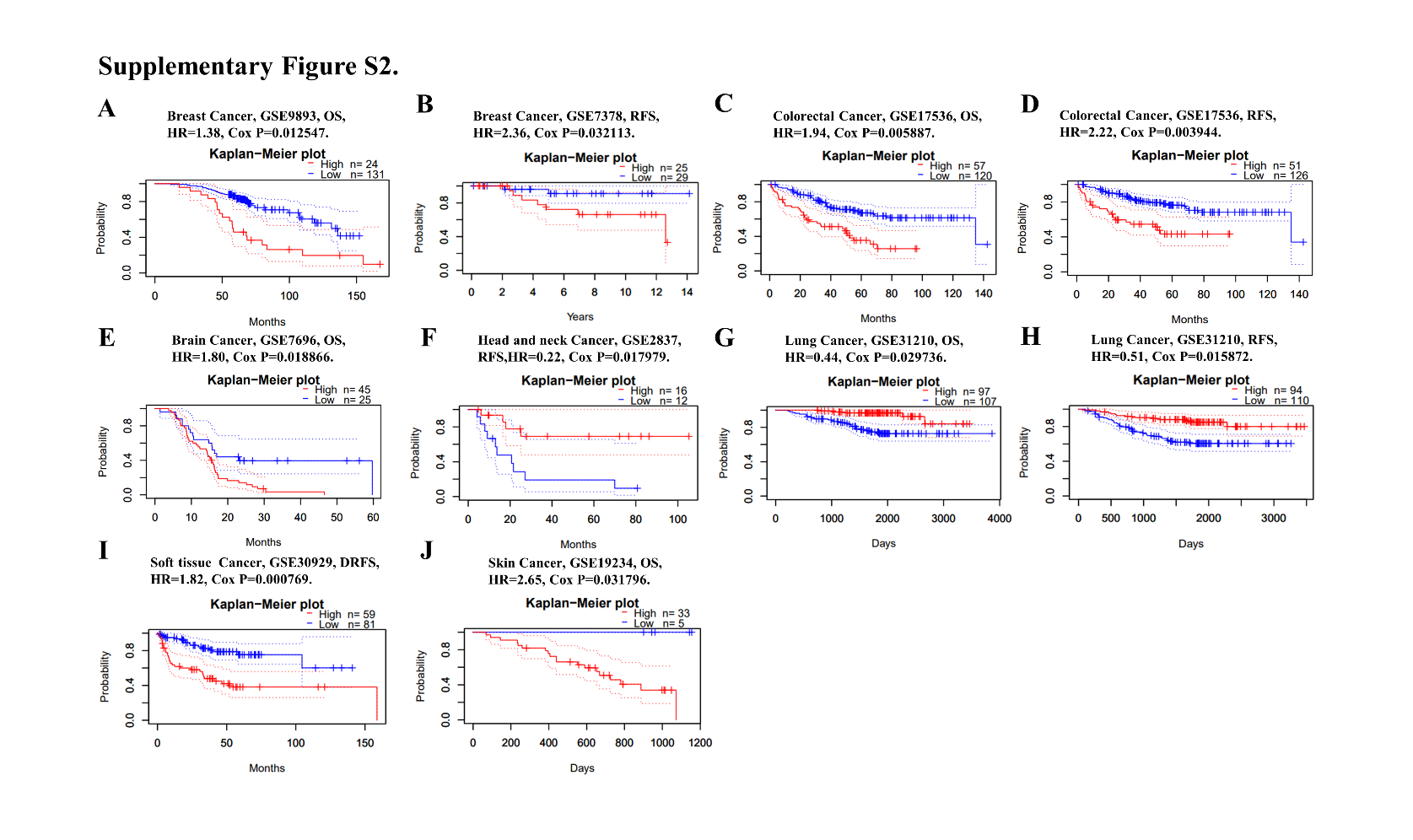


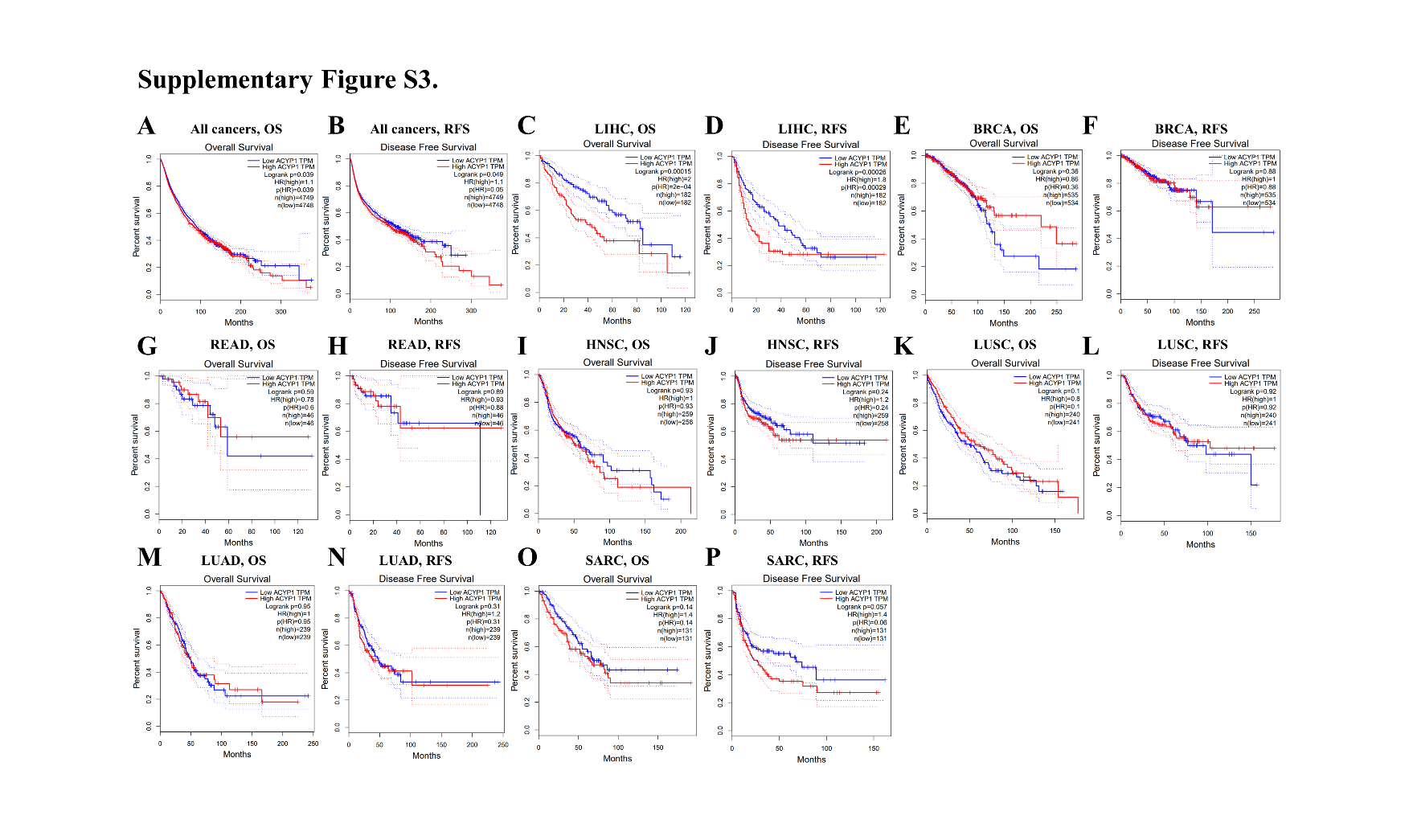


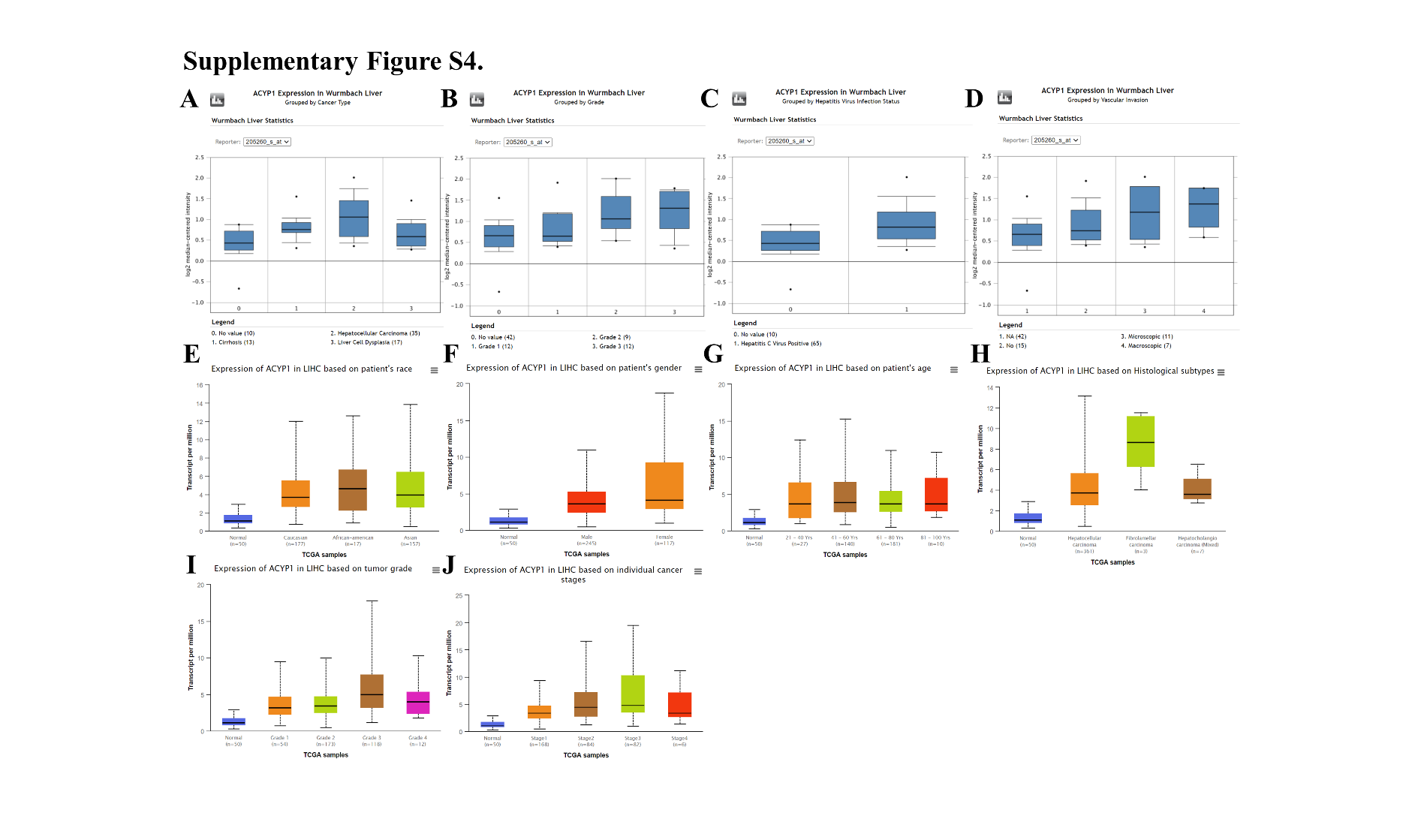


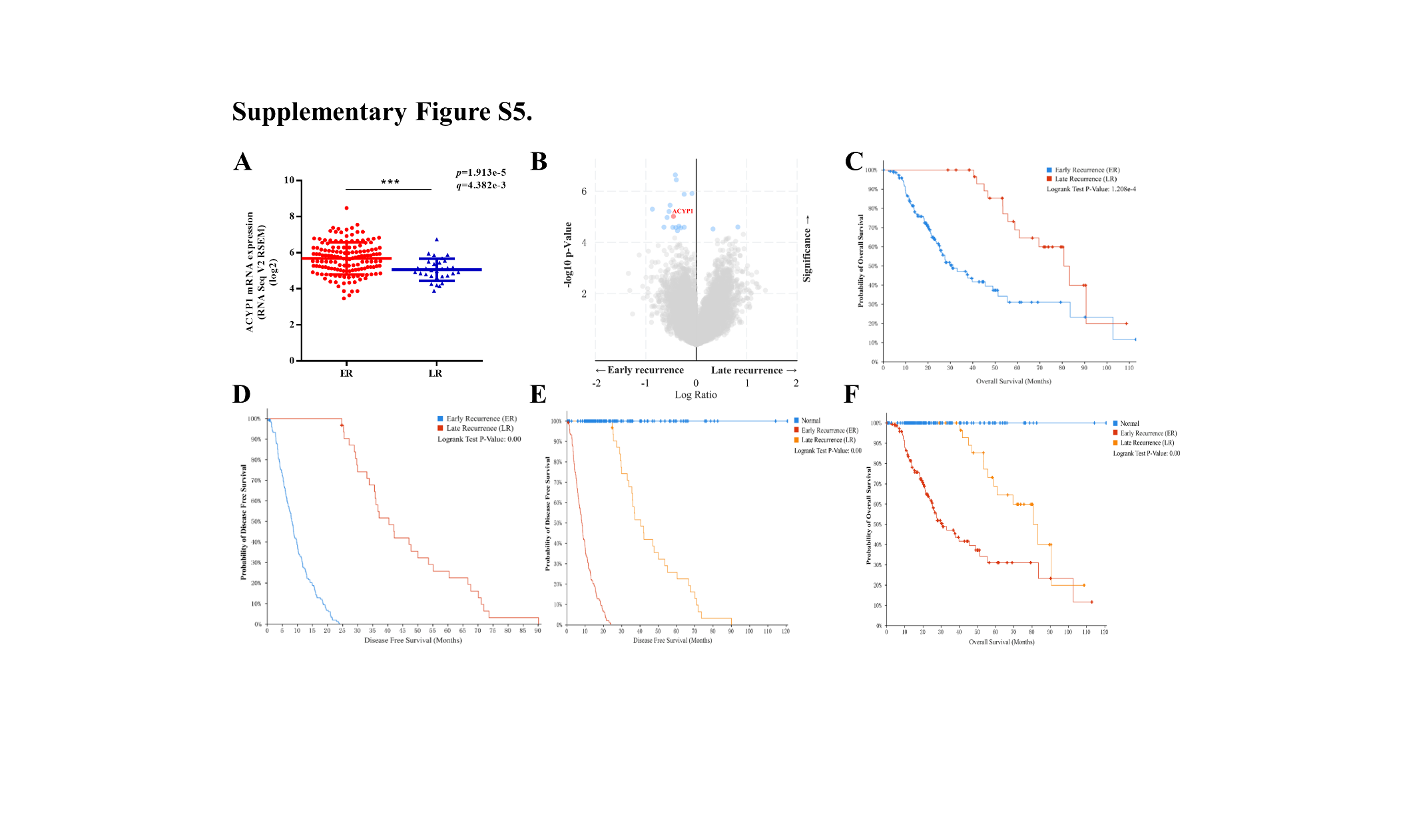


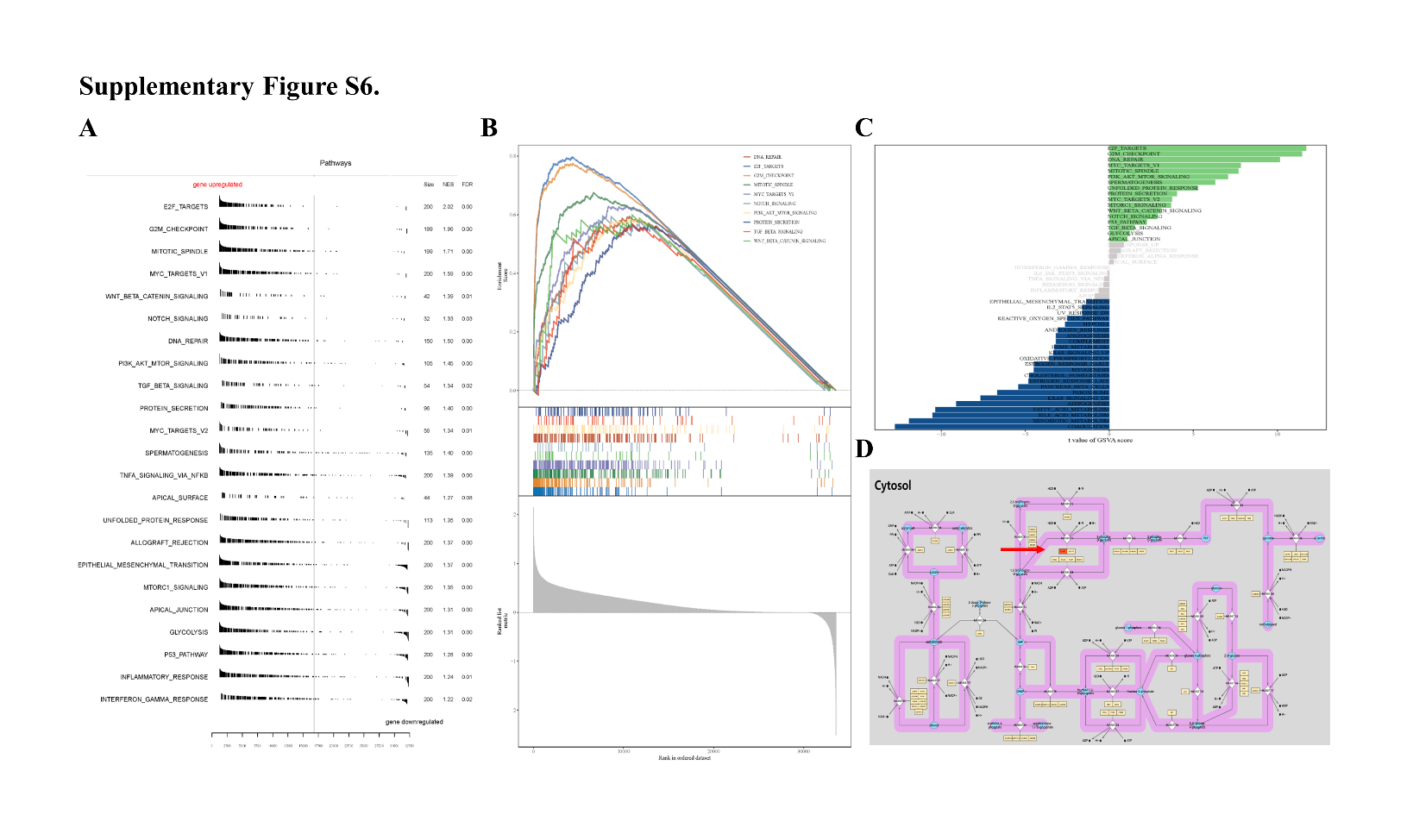


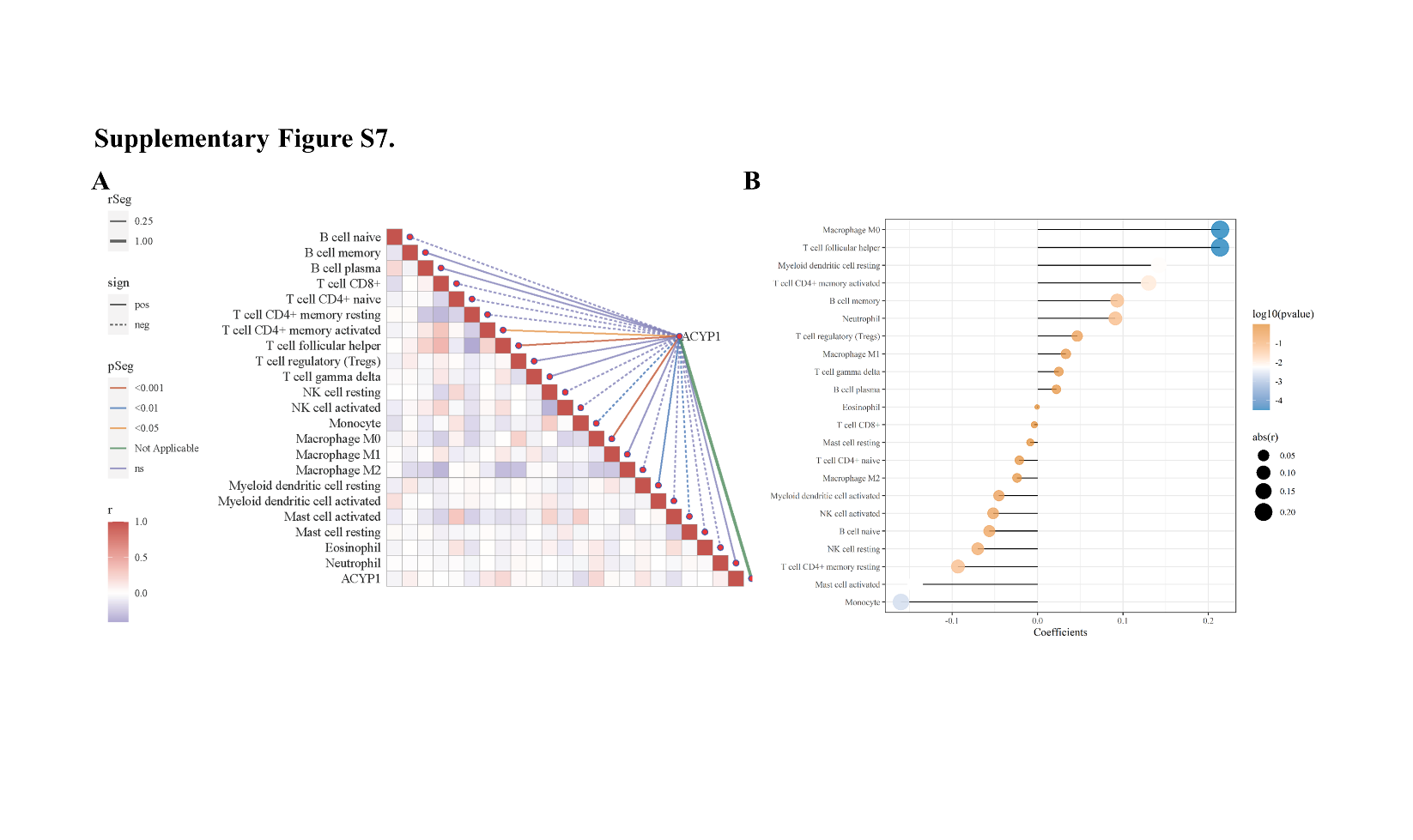


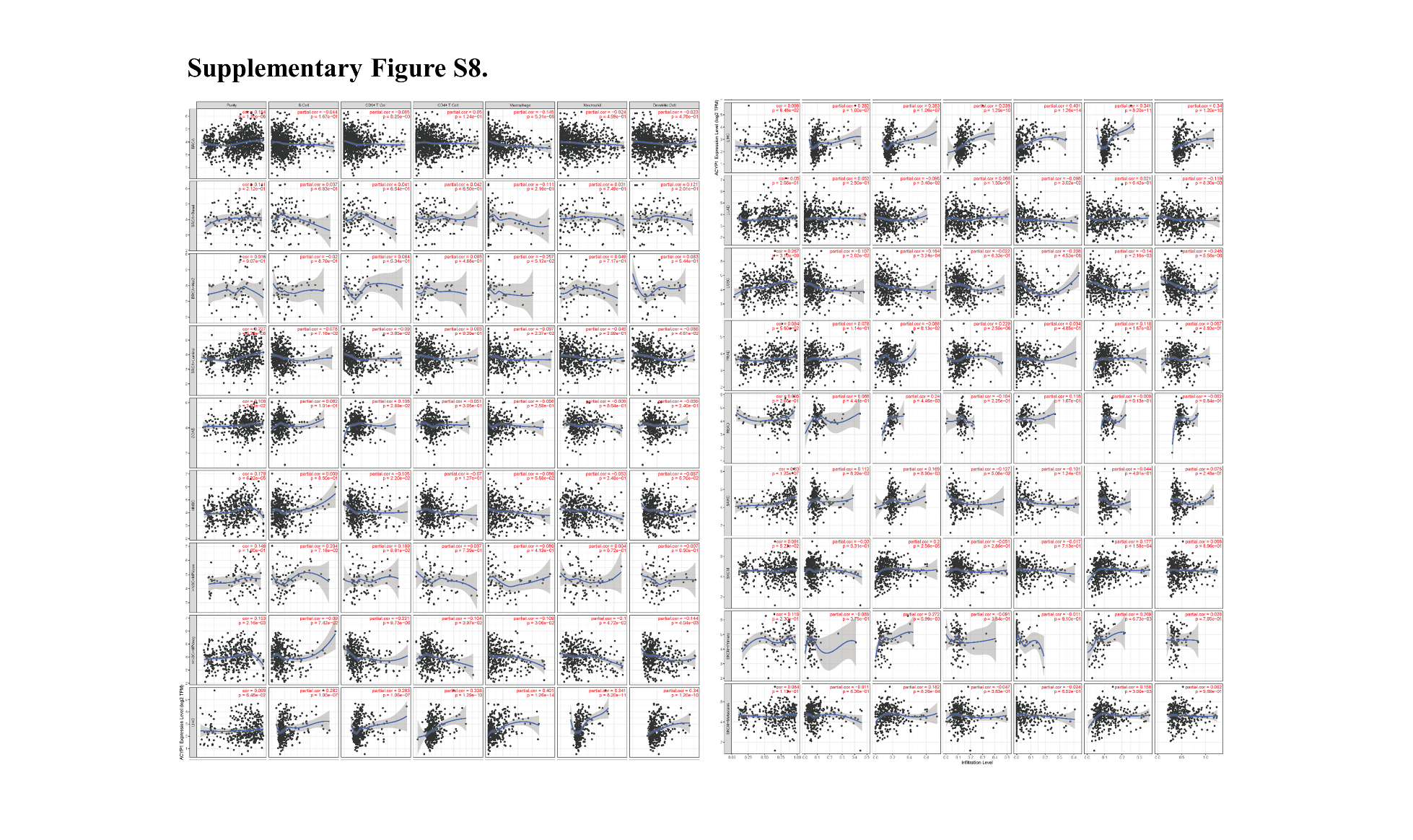


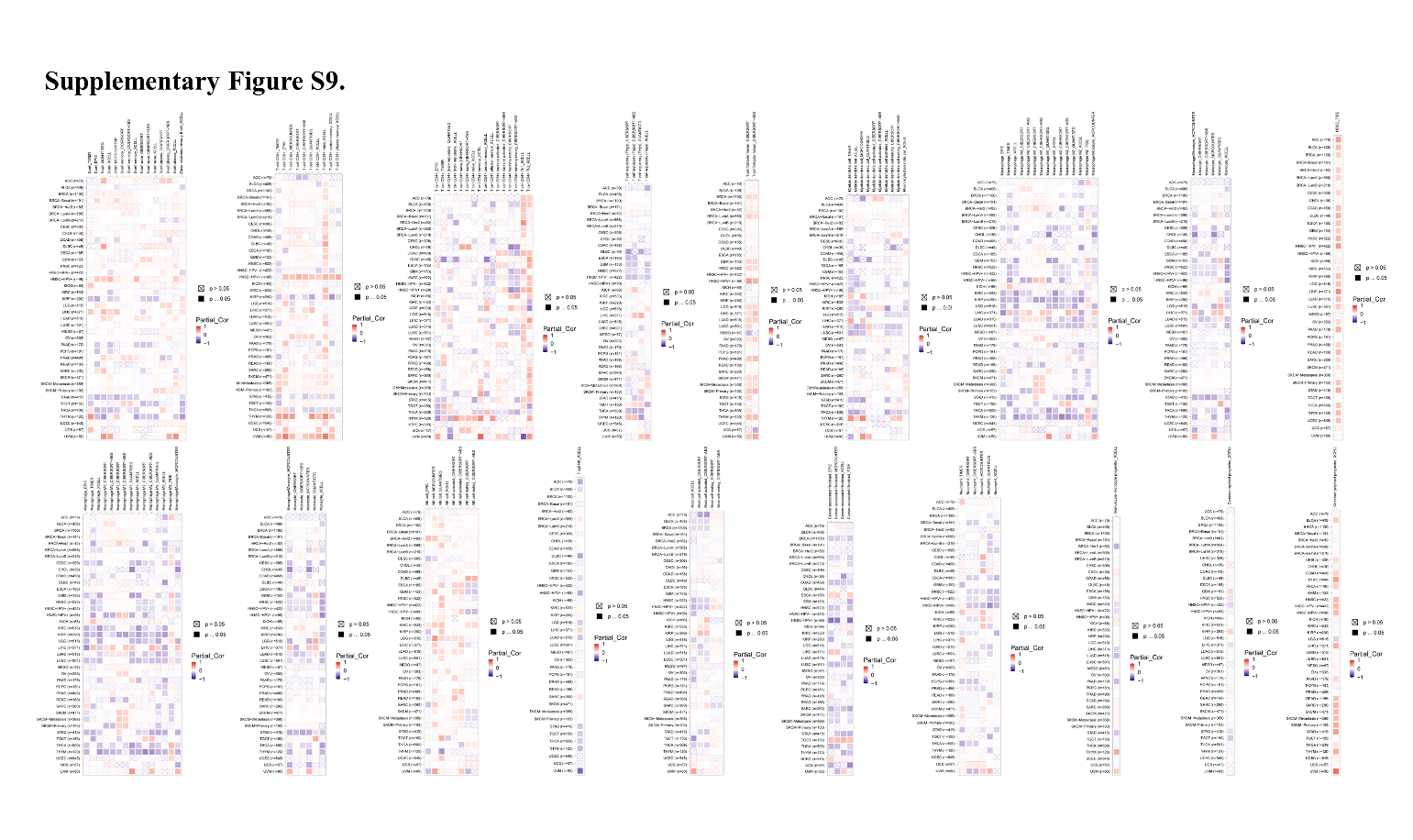


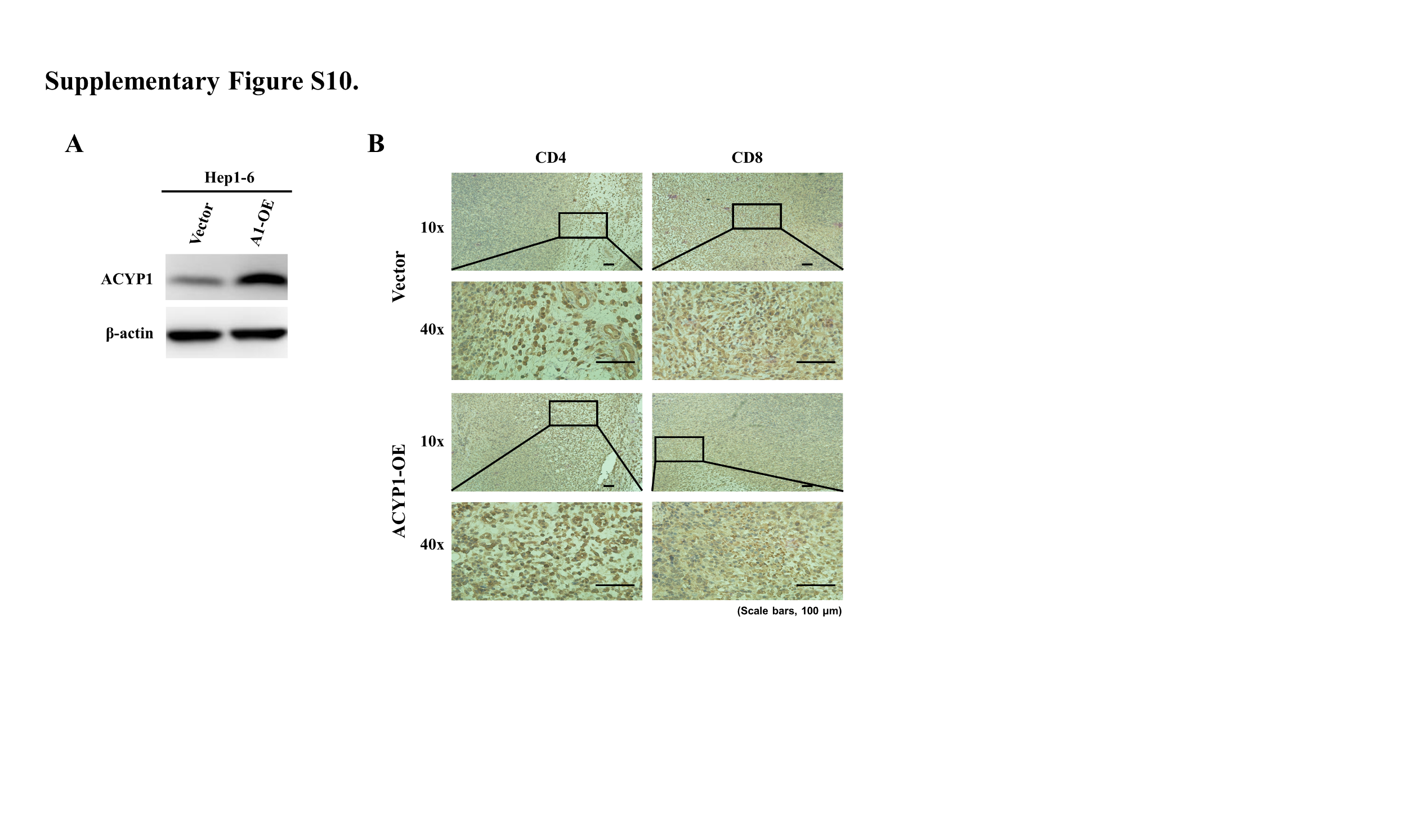


**Supplementary Figures:**

**Supplementary Figure S1.** The transcription levels of ACYP1 in human cancers. **(A)** The mRNA expression of ACYP1 between tumor and normal tissues was assessed from TCGA. **(B)** Radar plot of A1 expression in tumor and normal tissues from TCGA. **(C)** Radar plot of A1 expression in tumor and normal tissues from TCGA and GTEx.

**Supplementary Figure S2.** The survival of patients with high and low expression of ACYP1 across cancers in the PrognoScan database. **(A)** OS (n=155) in breast cancer cohort GSE9893. **(B)** RFS (n=54) in breast cancer cohort GSE7378. **(C)** OS (n=177) in colorectal cancer cohort GSE17536. **(D)** RFS (n=177) in colorectal cancer cohort GSE17536. **(E)** OS (n=70) in brain cancer cohort GSE7696. **(F)** RFS (n=28) in head and neck cancer cohort GSE2837. **(G)** OS (n=204) in lung cancer cohort GSE31210. **(H)** RFS (n=204) in lung cancer cohort GSE31210. **(I)** DRFS (n=140) in soft tissue cancer cohort GSE30929. **(J)** OS (n=38) in skin cancer cohort GSE19234. OS, overall survival; RFS, relapse-free survival; DRFS, distant recurrence-free survival.

**Supplementary Figure S3.** The survival of patients with high and low expression of ACYP1 across cancers in the GEPIA database. OS and RFS of all cancer patients **(A, B)**. OS and RFS of LIHC **(C, D)**, BRCA **(E, F)**, READ **(G, H)**, HNSC **(I, J)**, LUSC **(K, L)**, LUAD **(M, N)**, and SARC **(O, P)** patients. OS, overall survival; RFS, relapse-free survival.

**Supplementary Figure S4.** Association of ACYP1 expression and different clinical characteristics in LIHC. Association of ACYP1 mRNA expression and cancer type **(A)**, grade **(B)**, hepatitis virus infection status **(C)**, and vascular invasion **(D)** of LIHC patients in Wurmbach mixed liver dataset. Association of ACYP1 mRNA expression and patient’s race **(E)**, patient’s gender **(F)**, patient’s age **(G)**, histological subtypes **(H)**, tumor grade **(I)**, and cancer stages **(J)** in UALCAN dataset.

**Supplementary Figure S5.** Correlation of ACYP1 mRNA expression with early and late recurrence in LIHC. **(A)** Differential expression of ACYP1 in LIHC patients with early and late recurrence. **(B)** Volcano plot of differentially expressed genes in LIHC patients with early and late recurrence. Red indicates the location of the ACYP1 gene. Survival curves of patients with early and late recurrence in LIHC with OS **(C)** and DFS **(D)**. Survival curves of patients with normal, early and late recurrence in LIHC with OS **(E)** and DFS **(F)**.

**Supplementary Figure S6.** Significant pathways influenced by ACYP1. **(A)** Relationships between ACYP1 and Hallmark pathways in LIHC from TCGA analyzed by GSEA. **(B)** The top ten signaling pathways with significant positive correlations with ACYP1 in LIHC from TCGA analyzed by GSEA. **(C)** Relationships between ACYP1 and Hallmark pathways in LIHC from TCGA analyzed by GSVA. **(D)** The pathways of ACYP1 involved in glycolysis from The Human Protein Atlas. (NSE≥1.0, FDR<0.25).

**Supplementary Figure S7.** Correlation of ACYP1 expression with the infiltration of various immune cells in LIHC via using CIBERSORT. **(A)** Heat map plot of correlation between ACYP1 and immune cells infiltration. **(B)** Lollipop plot of correlation between ACYP1 and immune cells infiltration.

**Supplementary Figure S8.** Correlation of ACYP1 expression with the infiltration of various immune cells in all other tumors by using TIMER 1.0.

**Supplementary Figure S9.** Correlation of ACYP1 expression with the infiltration of various immune cells in all other tumors by using TIMER 2.0.

**Supplementary Figure S10.** (A) Validation of ACYP1 expression in ACYP1-overexpressing stable cell lines by Western Blotting. (B) Representative IHC staining of CD4+ and CD8+ T cells in subcutaneous Hep1-6 tumors from vector mice and ACYP1 overexpression mice at 100x and 400x magnification.

**Supplementary Tables:**

**Supplementary Table S1.** The transcription levels of ACYP1 in various cancer versus normal tissues in Oncomine.

**Supplementary Table S2**. Pathways influenced by ACYP1 by GSEA. The table include two sheets, which showed separately the GSEA KEGG and GSEA Hallmark output.

**Supplementary Table S3.** Pathways influenced by ACYP1 by GSVA. The table include two sheets, which showed separately the GSVA KEGG and GSVA Hallmark output.

**Supplementary Table S4**. The relationship between ACYP1 and immune cells in LIHC via using CIBERSORT. The table include two sheets, which showed separately the heat map plot and lollipop plot analysis.
